# Exploration of Nitrate Reductase Metabolic Pathway in* Corynebacterium pseudotuberculosis*

**DOI:** 10.1155/2017/9481756

**Published:** 2017-02-20

**Authors:** Sintia Almeida, Cassiana Sousa, Vinícius Abreu, Carlos Diniz, Elaine M. S. Dorneles, Andrey P. Lage, Debmalya Barh, Vasco Azevedo

**Affiliations:** ^1^Institute of Biologic Sciences, Federal University of Minas Gerais, 31270-901 Belo Horizonte, MG, Brazil; ^2^Center for Nuclear Energy in Agriculture, University of Sao Paulo, 13400-970 Piracicaba, SP, Brazil; ^3^Escola de Veterinária, Federal University of Minas Gerais, Belo Horizonte, MG, Brazil; ^4^Departamento de Medicina Veterinária, Universidade Federal de Lavras, Lavras, MG, Brazil; ^5^Centre for Genomics and Applied Gene Technology, Institute of Integrative Omics and Applied Biotechnology (IIOAB), Nonakuri, Purba Medinipur, West Bengal, India

## Abstract

Based on the ability of nitrate reductase synthesis,* Corynebacterium pseudotuberculosis *is classified into two biovars: Ovis and Equi. Due to the presence of nitrate reductase, the Equi biovar can survive in absence of oxygen. On the other hand, Ovis biovar that does not have nitrate reductase is able to adapt to various ecological niches and can grow on certain carbon sources. Apart from these two biovars, some other strains are also able to carry out the reduction of nitrate. The enzymes that are involved in electron transport chain are also identified by in silico methods. Findings about pathogen metabolism can contribute to the identification of relationship between nitrate reductase and the* C. pseudotuberculosis* pathogenicity, virulence factors, and discovery of drug targets.

## 1. Introduction

*Corynebacterium pseudotuberculosis* is a Gram-positive facultative intracellular pathogen [1–11].* C. pseudotuberculosis* can be classified into two biovars, based on their ability to convert nitrate to nitrite. The nitrate-positive biovar is Equi, which causes ulcerative lymphangitis in equines, while the nitrate-negative biovar is known as Ovis, which is the etiologic agent of caseous lymphadenitis in small ruminants [9]. Both diseases are globally distributed and cause large economic losses to goat, sheep, horse, and cattle farmers.

The nitrate reduction is associated with the bacterium's ability to breathe in the absence of oxygen and having two different metabolic pathways, (1) respiratory nitrate reductase and (2) dissimilatory nitrate reduction. In the first pathway, the denitrification process takes place where the nitrate is sequentially reduced to nitrite, nitric oxide, nitrous oxide, and finally to dinitrogen [12, 13]. In the second pathway nitrate is directly converted into ammonia, which is secreted from the cell; this process can be performed by organisms with the* nrf* gene. This is a less common method of nitrate reduction than denitrification in most ecosystems [12–14]. There are four classes of nitrate reductases, one in eukaryotes and three are in prokaryotes. Prokaryotic nitrate reductases include a class of assimilatory enzymes and two classes of respiratory enzymes; all contain a guanylate molybdenum cofactor but differ in their substructures, cellular location, and requirement for cofactor. Variability among enzyme is also found into the classes [12, 13].

Among the Actinobacteria, respiratory nitrate reductase (Nar) is observed [15–18]. This class of enzymes is mostly membrane-bound and is having three different subunits: respiratory nitrate reductase from* Pseudomonas stutzeri* [19, 20], nitrate reductase A, and nitrate reductase Z from* Escherichia coli* K-12 [21–23]. In* Corynebacterium glutamicum* and* Mycobacterium tuberculosis* the nitrate reduction reaction is catalyzed by the operons* narKGHJI* and* narGHJI*, respectively [15, 16]. In these organisms, the first step in nitrate assimilation is nitrate reduction (NO_3_^−^) to nitrite (NO_2_^−^) and the second step is nitrite reduced (N_3_^−^) to ammonia (NH_4_^+^) [15, 16].

Nitrate respiration is associated with virulence and adoption of pathogenic organisms. In* Brucella suis* it helps in intramacrophagic multiplication, in* M. bovis* it allows survival inside the host, and in* M. tuberculosis* it confers resistance to stress [17, 24, 25]. However, the role of nitrate respiration is not known in* C. pseudotuberculosis*. We presume that Nar may be associated with pathogenicity in* C. pseudotuberculosis* biovar Equi, providing a generate energy pathway when it grows under oxygen deprived condition allowing the survival of* C. pseudotuberculosis* inside the host.

With the increase in genome sequencing projects, understanding of the biological capabilities of the organisms has also increased. The availability of the annotated genomes allows the information of metabolic pathways to be interpreted and used for computational reconstruction at genomic scale. Thus the reconstruction of metabolic pathways through comparative analysis can help identify potential targets and find the mechanisms that cause a disease.

In the present study, the genomes of* C. pseudotuberculosis* strains deposited in GenBank were used to identify the genes involved in the nitrate reduction pathway; a comparative genomic study was performed on 15* C. pseudotuberculosis* strains of the biovars Ovis and Equi to identify the molecular bases of nitrate reduction in each* C. pseudotuberculosis* biovar Equi. Thus, we hope that understanding of Nar's role in* C. pseudotuberculosis* can help in identifying potent targets both for the development of more effective diagnostics and therapeutics and for the treatment and prevention of disease.

## 2. Material and Methods

### 2.1. Identification of Nitrate Reductase Genes, Metabolic Pathways, and Nitrate Reduction Enzymes

*C. pseudotuberculosis* genomes Table 1 were obtained from the NCBI GenBank. The sequence and arrangement analysis of nitrate reductase genes in* narKGHJI* operon was performed using the Artemis software (http://www.sanger.ac.uk/Software/Artemis) and manual curation based annotations are performed. Pathway Tools software developed by SRI International [45] and MetaCyc database [46] were used for computational prediction of metabolic pathways in* C. pseudotuberculosis*.

### 2.2. *narKGHJI *Operon Analysis

The families of proteins as well as the conserved protein domains present in the operon* narKGHJI* and adjacent genes were analyzed using InterProScan database (https://www.ebi.ac.uk/interpro). Prediction of transmembrane helices in protein was performed using TMHMM Server v. 2.0 (http://www.cbs.dtu.dk/services/TMHMM/). The similarity analysis of genes involved in the nitrate reduction pathway was performed using the UniProtKB database in category BLASTp. Clustal Omega was used to multiple sequence alignment between operon* narKGHJI *of* C. pseudotuberculosis* biovar Equi and homologous sequences of multiple organisms (http://www.ebi.ac.uk/Tools/msa/clustalo/), and Jalview was used to analyze similarity (http://www.jalview.org/) among these sequences.

## 3. Results and Discussion

### 3.1. Correlation between Nitrate Reduction Phenotype and Genotype

Nineteen* C. pseudotuberculosis* strains that had genome information in GenBank are presented Table 1. To perform the phenotyping analysis, 18 strains that were sequenced in our lab were used. All these strains were biochemically tested in order to verify their ability of nitrate reduction; see Table 1. Among the* C. pseudotuberculosis *strains, 31, 258, 262, and MB20 strains showed concordance between phenotypic and genotypic results. Strains 1/06-A, 316, 162, and CIP52.97 are found positive phenotype for reduction of nitrate; however they do not have genes involved in nitrate reduction in their genome.

### 3.2. Genes Involved in Respiratory Nitrate Reductase Metabolic Pathway

**Figure 1:**
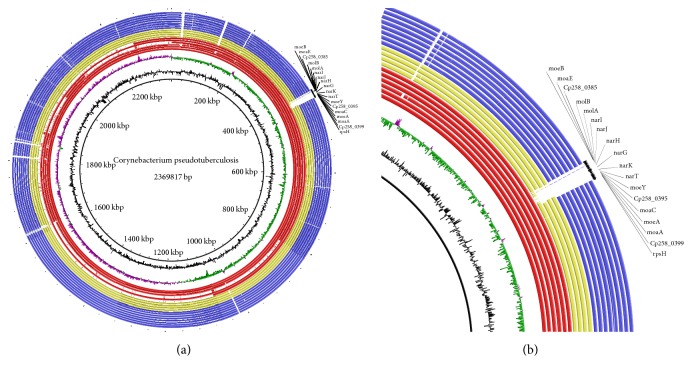
Graphical circular map showing BLAST between nineteen* C. pseudotuberculosis* strains. (a) From center to the outside: GC content in black and GC skew in pink and green; in red and yellow Equi strains and in blue Ovis strains. From the inner to outer circle on (a) and (b): the biovar* Equi* strains are the following: 258, 31, 262, MB20, E19, CCUG27541, CIP52.97, 1/06-A, 316, and 162, and the biovar Ovis strains are the following: 1002, C231, FRC41, I19, PAT10, 42/02-A, 3/99-5, 267, and P54B96. (b) Zoom to the nitrate* locus*, absent in the biovar* Equi* strains CIP52.97, 1/06 –A, 316, and 162 and all biovar Ovis strains.

We first compared the autoassigned pathways by the Pathologic software with the pathways in databases such as KEGG (http://www.genome.jp/kegg/) and resolved possible discrepancies using manual curation. Comparative genome analysis revealed that* C. pseudotuberculosis *biovar Equi possess* narKGHJI* gene clusters that participate via the respiratory anaerobic process of the nitrate reduction similarly to* E. coli *(Figure S8) [47]. The* C. pseudotuberculosis narKGHJI* gene cluster showed great similarity with the protein sequences found in other Actinomycetes, such as* C. diphtheriae*,* C. glutamicum,* and* M. tuberculosis* (Table 2). All biovar Ovis strains do not present any gene of the* narKGHJI* operon in their genomes (Figure 1).

**Figure 2:**
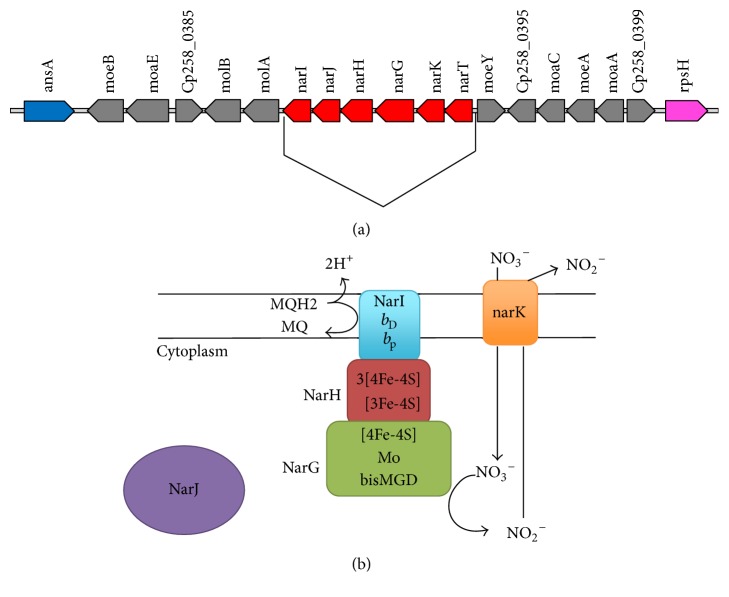
Nitrate* locus* from* C. pseudotuberculosis* biovar Equi. (a) This* locus *contains the following: genes encoding the nitrate reductase* narK*,* narG*,* narH*,* narJ*, and* narI*, and the genes encoding the molybdopterins* moeB, moaE, molB, molA, moeY, moaC, moeA,* and* moaA*. Insertion between* ansA* and* rpsH*: genes encoding the nitrate reductase are lacking in nitrate-negative* C. pseudotuberculosis *biovar Ovis strains. Arrows represent open reading frames and their orientations. (b) Bacterial nitrate respiration. The NarG and NarH bind to the membrane via the interaction between a hydrophobic patch of NarH and NarI which is buried within the membrane. The NarJis a chaperone that is involved in folding, maturation, and molybdenum cofactor insertion of nitrate reductase.

The nitrate locus that contains* narKGHJI* operon in* C. pseudotuberculosis* is composed of gene cluster encoding the molybdopterins* moeB, moaE, molB, molA, moeY, moaC, moeA,* and* moa *(Figure 2). Molybdopterin is a cofactor that is indispensable for activity of nitrate reductase [48]. The organization and conservation of the* narKGHJI* gene cluster was assessed among* C. pseudotuberculosis *and* C. diphtheriae*,* C. glutamicum, M. tuberculosis* and,* E. coli. *The analysis showed that* narKGHJI* gene cluster is conserved among these species; however gene orientation of the five genes is only conserved among* C. pseudotuberculosis, C. diphtheriae,* and* C. glutamicum. *In addition, in* C. pseudotuberculosis* we found* narT* gene is located at the upstream of the* narKGHJI* cluster. The* narT* gene encodes a nitrate transporter and is not present in* E. coli* and* C. glutamicum*.

### 3.3. Enzymes Involved in Electron Transport Chain

We found conserved functional domains of the genes present in the* narGHI *complex (involved in electron transport chain) in* C. pseudotuberculosis *biovar Equi (Table 3). To infer the functionality of* narGHI* complex, we used tridimensional structure from* E. coli* (PDB accession 1Q16) as a model for* nar* genes, as the structure of these enzymes is resolved in these bacteria.

In the* narGHI* complex in Figure 2, the* narG* gene is a member of a superfamily of enzymes that use a Mo-bisMGD cofactor (bisMGD) for their catalytic activity. Residues present in the active site of the molybdenum atom (Mo) are highly conserved in NarG subunits of Gram-negative and Gram-positive bacterial species [21]. The NarH belongs to the superfamily of electron transfer subunit (ferredoxins) of bacterial oxidoreductases. NarH has three clusters [4Fe-4S] (FS1, FS2, and FS3) and a [3Fe-4S] cluster (FS4) [21, 42, 44]. The general structure of NarH in* E. coli* is composed of a central region of a linker region that is inserted between the two subdomains in coordination of the iron-sulfur clusters and forms an extended connection between the NarG and NarH subunits and an extended C-terminus. In* C. pseudotuberculosis* it was observed that the nine cysteine residues responsible for coordinating three clusters [4Fe-4S] and one cluster [3Fe-4S] are conserved in all analyzed sequences. NarI is the transmembrane subunit that anchors NarGH to the cytoplasmic side of the membrane and provides the connection between the quinone and the site of oxidation, referred to as “Site Q.” This subunit coordinates two hemes, one of which is located towards the side of cytoplasmic end of NarI and is referred to as the proximal heme (heme *b*_P_), while the other is located towards the periplasmic side of the same and is referred to as the distal heme (heme *b*_D_). Such hemes act as mediators in electron transfer from Site Q to the clusters [Fe-S] in NarH. NarI of* C. pseudotuberculosis* presented five transmembrane helices S1 Figure, which is in agreement with the literature when compared to the* E. coli* crystal structure [49, 50]. This C-terminal segment presents highly conserved residues in the family NarI proteins and also is present in* C. pseudotuberculosis. *It is interesting to note that NarI is considerably preserved in relation to its primary sequence between orthologous proteins found in species of the* Corynebacterium* genus, particularly in relation to* C. diphtheriae*, which has 96% identity and 98% similarity. These results suggest that the enzymatic complex* narGHI* is probably very similar with respect to its three dimensional structure among the species of* C. pseudotuberculosis* biovar Equi and* C. diphtheriae* in view of the high identity found among NarG, NaH, and NarI subunits of these species. The* C. pseudotuberculosis* biovar Equi presents 48% and 43% identity in relation to the orthologous proteins from* M. tuberculosis* and* B. subtilis*, respectively.

### 3.4. NarJ (Molybdenum-Cofactor-Assembly Chaperone)

The NarJ is a chaperone that is involved in folding, maturation, and molybdenum cofactor insertion of nitrate reductase in* E. coli* [51, 52]. The similarity values found between* C. pseudotuberculosis *biovar Equi and other sequences of orthologous proteins by BLAST, including the genus* Corynebacterium*, were slightly lower than those found in other proteins (NarG, NarH, and NarI) that form the three subunits of the enzyme nitrate reductase (Table 2). However,* narJ *remained conserved between* C. pseudotuberculosis *biovar Equi strains, and results of biochemical tests (Table 1) indicate that its function has remained preserved, since NarJ is important for nitrate reductase expression in others organisms [53].

### 3.5. Nitrate and Nitrite Transporters

The nitrate and nitrite export is mediated by nitrate and nitrite transporters that are members of the major facilitator superfamily (MFS) [54]. In bacteria such carriers are the* narK* family represented by NarK and NarU proteins in* E. coli *that transport both nitrate and nitrite [47]. However, NarK is expressed at higher levels than NarU and it remains unclear till date if NarK is a nitrate/nitrite antiporter or symporter [55]. In* C. pseudotuberculosis *biovar Equi, we found that the two genes* narK* and* narT* are responsible for coding two probable nitrate and nitrite carriers and in* C. pseudotuberculosis *biovar Equi is present at the upstream of the* narGHJI *operon (Figure 2). Both proteins are found to be composed of 12 transmembrane helices similar to other members of the superfamily of bacterial transporters; see S2 and S3 Figures. In* C. glutamicum* the* narK* is in close proximity to* narG, narH, narJ, *and* narI*, which together form the* narKGHJI *operon [56], but in* E. coli*,* narGHJI* operon is transcribed individually and* narT* is upstream of the operon* narKGHJI *[57]. Since we did only the in silico analysis, we cannot state the precise transcription and regulation of these genes in vivo. We presume that this* narKGHJI* cluster in* C. pseudotuberculosis *biovar Equi may be regulated together as found in* C. glutamicum*. However, experimental confirmation is required.

### 3.6. Nitrite Reductase in* C. pseudotuberculosis*

Genes in* C. pseudotuberculosis* are found to encode two different types of nitrite reductase enzymes. One is the nitric oxide-forming enzyme that is encoded by* nirK* gene and shown to be a copper dependent enzyme. The second groups of enzymes are ammonium nitrite reductase that are encoded by* nrfA* gene that encodes catalytic subunit and* nrfH* that encodes for a membrane associated electron transfer subunit. In all* C. pseudotuberculosis *strains we found NrfA and NrfH subunits of nitrite reductase NrfAH complex that catalyze the reduction of nitrite to ammonium. Beside* C. pseudotuberculosis*, such genes are found only in* C. ulcerans* and are absent in all other species from this genus. In* C. pseudotuberculosis*, the two genes* nrfA* and* nrfH* are located in a “cluster” of genes, S4 Figure, responsible for coding biogenesis system I proteins cytochrome c that appears to be a common feature among bacterial genomes.* C. pseudotuberculosis* also has a nitrite reductase enzyme likely dependent on copper (CuNIR). The gene for CuNIR is nirK that encodes a protein composed of 882 residues and is conserved in all strains of* C. pseudotuberculosis*; see Figure S5 of the Supplementary Material available online at https://doi.org/10.1155/2017/9481756. The sequence similarity analysis of* nirK* was performed using the BLAST and it was observed that such protein has an N-terminal region of about 500 amino acids, S6 Figure, compared to the most homologous proteins that have been experimentally well characterized [58–61]. Within the genus* Corynebacterium*, the CuNIR proteins are found to have similar sizes to that of* C. pseudotuberculosis*, such as* Corynebacterium durum* (WP_006063127.1) and* Corynebacterium vitaeruminis* (WP_051483598.1), with 40% identity between the sequences; see Table S1. Furthermore, it was found that the N-terminal region of about 500 residues is probably composed of 13 transmembrane helices in* C. pseudotuberculosis*, S7 Figure, which indicates that it can be a membrane associated protein. To verify the residues conservation, we used* G. kaustophilus* CuNIR proteins as a reference sequence because its crystal structure has recently been determined (pdb∣3WIA∣A). All residues responsible for binding to the copper atoms and the catalytic site were analyzed. It is observed that the C-terminal region is 100% conserved in* C. pseudotuberculosis*; see S6 Figure.

### 3.7. Reconstruction of* C. pseudotuberculosis* Biovar Equi Metabolic Network

**Figure 3:**
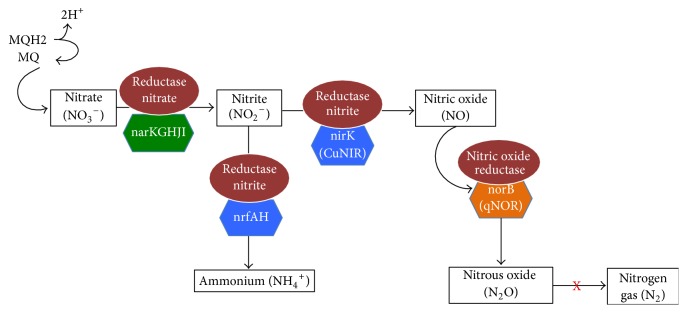
Model building of nitrate* reductase* from* C. pseudotuberculosis* biovar Equi. Showed here is respiratory nitrate reduction to nitrite; incomplete denitrification of nitrite in which nitrous oxide is the final product; and Nrf-dependent ammonification.

A metabolic network reconstruction is assembled piece-by-piece by compiling data on known enzymes, and annotated genome together with data from the literature is used to assemble a network reconstruction [62]. In* C. pseudotuberculosis* after the similarity analysis we observed that genomic sequences participating in the nitrate reduction pathway are conserved, and the residues were analyzed, indicating a possible conserved function. Thus we can infer that* C. pseudotuberculosis* biovar Equi is different from that occurring in* C. glutamicum*, where nitrate is excreted out of the cells as the major end product under the conditions of anaerobic respiration, and nitrate and nitrite are not used as sources of nitrogen for aerobic or anaerobic growth [18] so* C. pseudotuberculosis* biovar Equi has a likely route of denitrification composed of the nitrate reductase Nar, nitrite reductase dependent copper, and nitric oxide reductase quinol-dependent, and this is a way of incomplete denitrification, in which nitrous oxide is the final product, and, therefore, there is no presence of nitrous oxide reductase enzyme and subsequent reduction to nitrogen gas; see Figure 3. Furthermore* C. pseudotuberculosis* biovar Equi also shows the pathway of ammonification in dissimilatory nitrate reduction to ammonium, what has been proposed as catalyzed by the enzyme nitrate reductase Nar and nitrite reductase complex NrfAH, a membrane-associated cytochrome; see Figure 3.* E. coli* not only reduce nitrite to ammonia but also catalyze the reduction of nitrite to NO, which can be used as an electron acceptor for anaerobic respiration [63]. The presence of enzymes and pathways of nitrate reduction proposals was also compared between biovar Ovis and biovar Equi strains and with respect to enzymes, the main difference was the presence of the nitrate locus that contains the genes encoding the molybdopterin and the genes encoding the nitrate reductase and* a region* situated upstream of the* ansA* gene and this locus has a 19,606 pb length.

## 4. Conclusions 

In this study, we found that* C. pseudotuberculosis* strains 1/06-A, 316, 162, and CIP52.97 are able to do nitrate reduction but in their genomes they do not have genes associated with nitrate reduction, warning us about resequencing of these genomes. The* C. pseudotuberculosis* nitrate locus is composed of* narKGHJI *gene cluster and by genes encoding the molybdopterins* moeB*,* moaE*,* molB*,* molA*,* moeY*,* moaC*,* moeA*, and* moa* genes are similar to the other bacteria under Actinomycetes and all the biovar Ovis strains that lack the nitrate locus in their genomes. The* narGHI *complex in* C. pseudotuberculosis* biovar Equi shows conserved functional domains.* narK* and* narT* genes in* C. pseudotuberculosis* biovar Equi encode two putative nitrate and nitrite carriers, respectively. Finally, the* nirK, nrfA, *and* nrfH* encoding nitrite reductase enzymes are conserved in* C. pseudotuberculosis*. Therefore, the study of pathogen metabolism can contribute to the identification of pathogen virulence, discovery of drug targets, host response, and generating hypotheses for experimental investigation.

## Supplementary Material

Supplementary Figures 1 to 3 and Figure 7: Transmembrane helices prediction to NarI, NarK, NarT and NirK proteins, respectively, from C. pseudotuberculosis biovar Equi. Supplementary Figure 4: Transcription unit for the nrfHA genes into C. pseudotuberculosis biovar Equi. Supplementary Figure 5: Multiple sequence alignment of C. pseudotuberculosis biovar Equi nirK gene. Supplementary Figure 6: Amino acid sequence alignment of bacterial NirKs. Supplementary Figure 8: Comparative nitrogen metabolism between E.coli and C. pseudotuberculosis biovar Equi. In red pathway to E. coli, in green to C. pseudotuberculosis. Supplementary Table 1: Similarity sequence analysis of nirK gene of C. pseudotuberculosis and bacterial CuNiRs

## Figures and Tables

**Table 1:** Strains used in phenotypic and genotypic analysis regarding the nitrate reductase activity.

Strain	Biovar	Genome size (MB)	Nitrate test^a^	NCBI access	Ref
1002	Ovis	2.33511	Yes	NC_017300.1	[26]
C231	Ovis	2.32821	Yes	NC_017301.1	[26]
FRC41	Ovis	2.33791	Yes	NC_014329.1	[27]
I19	Ovis	2.33773	Yes	NC_017303.1	[28]
PAT10	Ovis	2.33532	Yes	NC_017305.1	[29]
42/02-A	Ovis	2.33761	Yes	NC_017306.1	[30]
3/99-5	Ovis	2.33794	Yes	NC_016781.1	[30]
267	Ovis	2.33763	Yes	NC_017462.1	[31]
P54B96	Ovis	2.33794	Yes	NC_017031.1	[32]
CIP5297	Equi	2.32059	Yes	NC_017307.1	[33]
1/06 -A	Equi	2.27912	Yes	NC_017308.1	[34]
316	Equi	2.31041	Yes	NC_016932.1	[35, 36]
258	Equi	2.36982	Yes	NC_017945.1	[37]
162	Equi	2.29346	Yes	NC_018019.1	[38]
31	Equi	2.38969	Yes	NC_017730.1	[39]
262	Equi	2.32575	Yes	NZ_CP012022.1	—
MB20	Equi	2.36309	Yes	JPUV01	[40]
E19	Equi	2.36796	Unknown	NZ_CP012136.1	—
CCUG27541	Equi	2.37942	Unknown	JPJB01	[41]

^a^Biochemical test.

**Table 2:** Similarity sequence analysis of *narKGHJI* gene clusters of *C. pseudotuberculosis*.

*C. pseudotuberculosis* protein	Species	Length (aa)	ID (%)	Similarity (%)	Access number/UniProtKB
Nitrate reductase alpha subunit (NarG)—1240aa	*Corynebacterium diphtheriae*	1,240	97	99	Q6NJA9
	*Corynebacterium glutamicum*	1,248	77	87	Q8NR68
	*Mycobacterium tuberculosis*	1,232	56	70	P9WJQ3
	*Escherichia coli*	1,247	46	64	P09152

Nitrate reductase beta subunit (NarH)—533aa	*Corynebacterium diphtheriae*	533	95	98	H2G670
	*Corynebacterium glutamicum*	531	79	89	S5Y1U5
	*Mycobacterium tuberculosis*	538	64	75	A0A049E025
	*Escherichia coli*	512	54	72	P11349

Nitrate reductase gamma subunit (NarI)—259aa	*Corynebacterium diphtheriae*	259	96	98	H2HVN2
	*Corynebacterium glutamicum*	259	75	89	S5YHX1
	*Mycobacterium tuberculosis*	241	48	68	Q7D8Q6
	*Escherichia coli*	225	28	51	P11350

Nitrate reductase chaperone (NarJ)—209aa	*Corynebacterium diphtheriae*	209	89	96	H2H309
	*Corynebacterium glutamicum*	228	63	80	Q6M5Z0
	*Mycobacterium tuberculosis*	206	37	49	Q7D8Q7
	*Escherichia coli*	236	30	46	P0AF26

Nitrate/nitrite transporter (NarK)—443aa	*C. diphtheriae*	443	93	97	H2HGI1
	*C. glutamicum*	445	66	78	I0LIN6
	*M. tuberculosis*	395	22	35	P9WJY7
	*E. coli*	463	33	51	P10903

**Table 3:** Residues analyzed regarding the conservation between the *narGHI* protein sequences of *E. coli* and *C. pseudotuberculosis*.

*E. coli*	*C. pseudotuberculosis*	Conservation (%)	Function	REF
*Residues NarG* ^*∗*^				
His49(H)	His55	100	Mo ion coordination	[42]
Cys53(C)	Cys 59	100	Mo ion coordination	[42]
Cys57(C)	Cys 63	100	Mo ion coordination	[42]
Cys92(C)	Cys 98	100	Mo ion coordination	[42]
Asp222 (D)	Asp 228	100	Mo-bisMGD ligand	[42]
Val 578 (V)	Val 584	100	Left relatively hydrophobic environment to allow binding within the active site, with one face of the side chain carboxylate exposed of Asp222. Optimal interaction with the Mo of the cofactor	[43]
Tyr 220 (Y)	Tyr 226	100	Left relatively hydrophobic environment to allow binding to within the active site, with one face of the side chain carboxylate exposed of Asp222. Allowing optimal interaction with the Mo of the cofactor	[43]
His1092(H)	His 1099	100	Form a hydrogen bond network that links the solvent interface with Asn^A52^ and could be important for structural integrity and/or proton delivery to the active site	[42]
His1098(H)	His 1105	100	Form a hydrogen bond network that links the solvent interface with Asn^A52^ and could be important for structural integrity and/or proton delivery to the active site	[42]
His1163(H)	His 1170	100	Form a hydrogen bond network that links the solvent interface with Asn^A52^ and could be important for structural integrity and/or proton delivery to the active site	[42]
Arg 94(R)	Arg 100	100	Forms a hydrogen bond with the Cys92 ligand of FS0	[43]
*Residues NarH* ^*∗*^				
Cys196 (C)	Cys 196	100	Binding of iron atoms of FS4 cluster [3Fe-4S]	[44]
Cys217(C)	Cys 217	100	Binding of iron atoms of FS4 cluster [3Fe-4S]	[44]
Cys223(C)	Cys 223	100	Binding of iron atoms of FS4 cluster [3Fe-4S]	[44]
Cys184(C)	Cys 184	100	Binding of iron atoms of FS3 cluster [4Fe-4S]	[44]
Cys187(C)	Cys 187	100	Binding of iron atoms of FS3 cluster [4Fe-4S]	[44]
Cys192(C)	Cys 192	100	Binding of iron atoms of FS3 cluster [4Fe-4S]	[44]
Cys 227(C)	Cys 227	100	Binding of iron atoms of FS3 cluster [4Fe-4S]	[44]
Cys26(C)	Cys 26	100	Binding of iron atoms of FS2 cluster [4Fe-4S]	[44]
Cys244(C)	Cys 244	100	Binding of iron atoms of FS2 cluster [4Fe-4S]	[44]
Cys247(C)	Cys 247	100	Binding of iron atoms of FS2 cluster [4Fe-4S]	[44]
Cys259(C)	Cys 259	100	Binding of iron atoms of FS2 cluster [4Fe-4S]	[44]
Cys16(C)	Cys 16	100	Binding of iron atoms of FS1 cluster [4Fe-4S]	[44]
Cys19(C)	Cys 19	100	Binding of iron atoms of FS1 cluster [4Fe-4S]	[44]
Cys22(C)	Cys 22	100	Binding of iron atoms of FS1 cluster [4Fe-4S]	[44]
Cys263 (C)	Cys 263	100	Binding of iron atoms of FS1 cluster [4Fe-4S]	[44]
*Residues NarI* ^*∗*^				
His66 (H)	His66	100	Iron atoms coordination of heme *b*_D_	[43]
His187 (H)	His190	100	Iron atoms coordination of heme *b*_D_	[43]
His56 (H)	His56	100	Iron atoms coordination of heme *b*_P_	[43]
His205 (H)	His208	100	Iron atoms coordination of heme *b*_P_	[43]
Arg112	Arg113	100	Hydrogen bond network electron transfer between the redox center *b*_P_, FS4, and NarG	[43]
Arg202	Arg205	100	Hydrogen bond network electron transfer between the redox center *b*_P_, FS4, and NarG.	[43]
Ser39	Ser39	100	Hydrogen bond network electron transfer between the redox center *b*_P_, FS4, and NarG.	[43]
Ser40	Ser40	100	Hydrogen bond network electron transfer between the redox center *b*_P_, FS4, and NarG.	[43]
Tyr213(Y)	Tyr216	100	Involved in electrostatic interactions and hydrogen bond, are involved in the formation of NarGHI heterotrimer	[43]
Arg216(R)	Arg219	100	Involved in electrostatic interactions and hydrogen bond, are involved in the formation of NarGHI heterotrimer	[43]
Arg222(R)	Arg225	100	Involved in electrostatic interactions and hydrogen bond, are involved in the formation of NarGHI heterotrimer	[43]
Ser201(S)	Ser204	100	A strong link at Ser201 of NarI allows distinctly shorter distances between the hemes in NarI than the distances observed between the hemes in the cytochrome bc1 complex	[43]

^*∗*^Shown is the position of the residues at *E. coli* and *C. pseudotuberculosis* sequences and as well as the function of each residue in the *E. colinarGHI* structure.
