# Supplementary material for: Exploration of Nitrate Reductase Metabolic Pathway in Corynebacterium pseudotuberculosis
Source: Int J Genomics. 2017 Feb 20;2017:9481756. doi: 10.1155/2017/9481756 (PMC5338063; doi:10.1155/2017/9481756)
Supplement: Supplementary file 1 — Supplementary Figures 1 to 3 and Figure 7: Transmembrane helices prediction to NarI, NarK, NarT and NirK proteins, respectively, from C. pseudotuberculosis biovar Equi. Supplementary Figure 4: Transcription unit for the nrfHA genes into C. pseudotuberculosis biovar Equi. Supplementary Figure 5: Multiple sequence alignment of C. pseudotuberculosis biovar Equi nirK gene. Supplementary Figure 6: Amino acid sequence alignment of bacterial NirKs. Supplementary Figure 8: Comparative nitrogen metabolism between E.coli and C. pseudotuberculosis biovar Equi. In red pathway to E. coli, in green to C. pseudotuberculosis. Supplementary Table 1: Similarity sequence analysis of nirK gene of C. pseudotuberculosis and bacterial CuNiRs [file 9481756.f1.pdf]

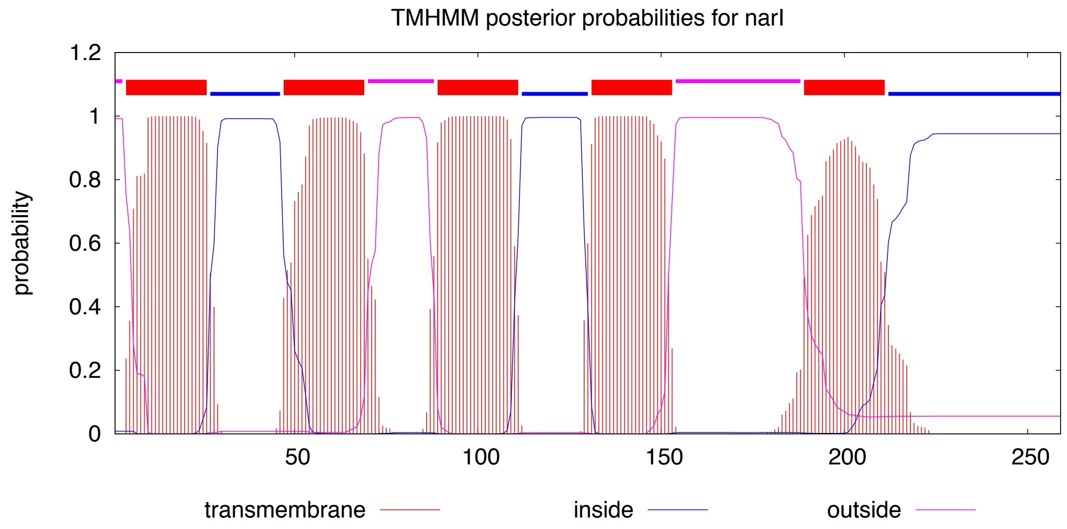

S1 Figure. Transmembrane helices in NarI protein from *C. pseudotuberculosis* biovar Equi.
NarI presented 5 transmembrane helices. The probability of a given amino acid being in transmembrane regions in the cytoplasmic or extracellular face is recorded in red, blue and pink, respectively. Prediction was performed with TMHMM 2.0 (http://www.cbs.dtu.dk/services/TMHMM/).

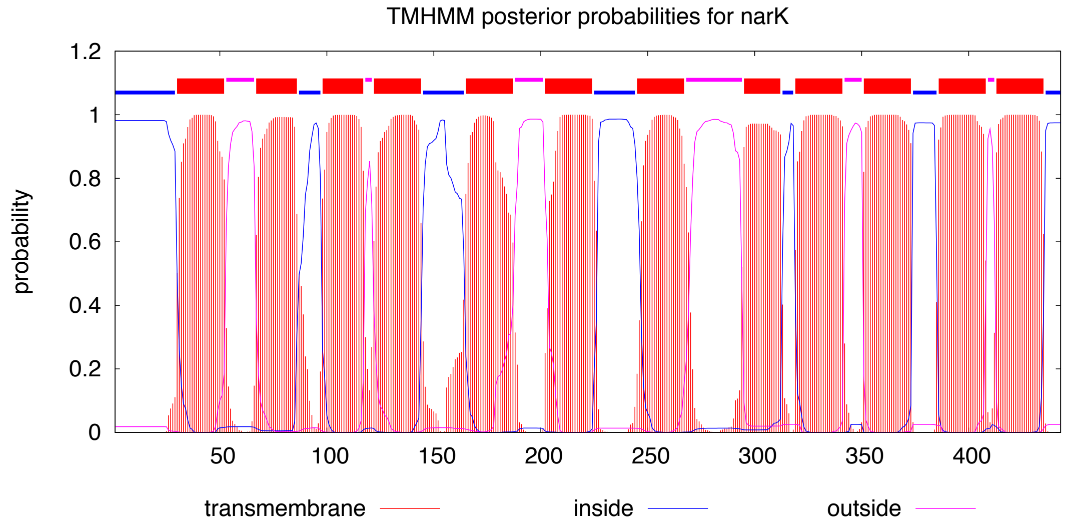

S2 Figure. Transmembrane helices in NarK protein from *C. pseudotuberculosis* biovar Equi.
NarK presented 12 transmembrane helices. Prediction was performed with TMHMM 2.0 (http://www.cbs.dtu.dk/services/TMHMM/).

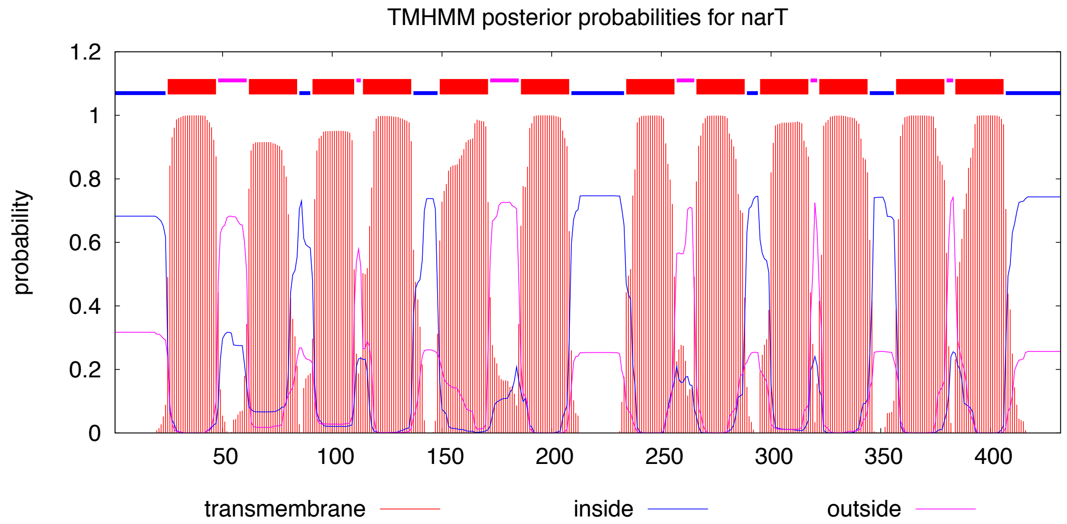

S3 Figure. Transmembrane helices in NarT protein from *C. pseudotuberculosis* biovar Equi.
NarT presented 12 transmembrane helices. Prediction was performed with TMHMM 2.0 (http://www.cbs.dtu.dk/services/TMHMM/).

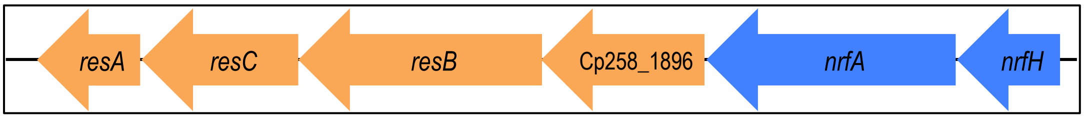

S4 Figure. Transcription unit for the *nrfHA* genes into *C. pseudotuberculosis* biovar Equi.
Genomic coordinate of 2046902 (*nrfH*) to 2052315 (*resA*). This region corresponds to 5414 pb lenght.

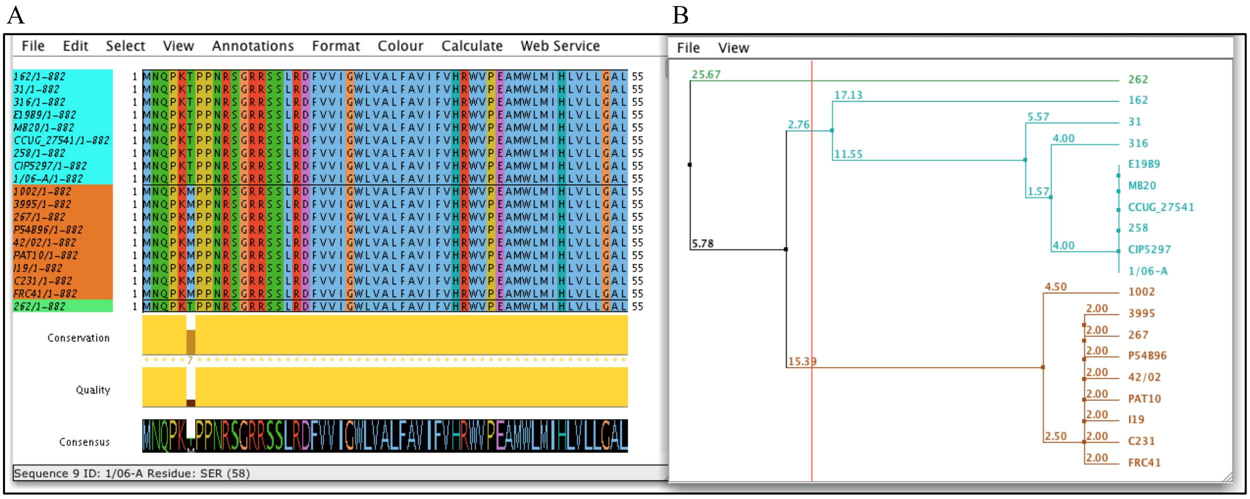

S5 Figure. Multiple Sequences alignment of *C. pseudotuberculosis* biovar Equi *nirK* gene.
In (A) multiple alignmet using Clustal Omega with default settings. Alignment was visualized in Jalview. In (B) Phylogenetic trees of *nirK* inferred by Jalview program with the average distance using BLOSUM62. Sequences for each species have been deposited in the GenBank databases, acess number Table 1.

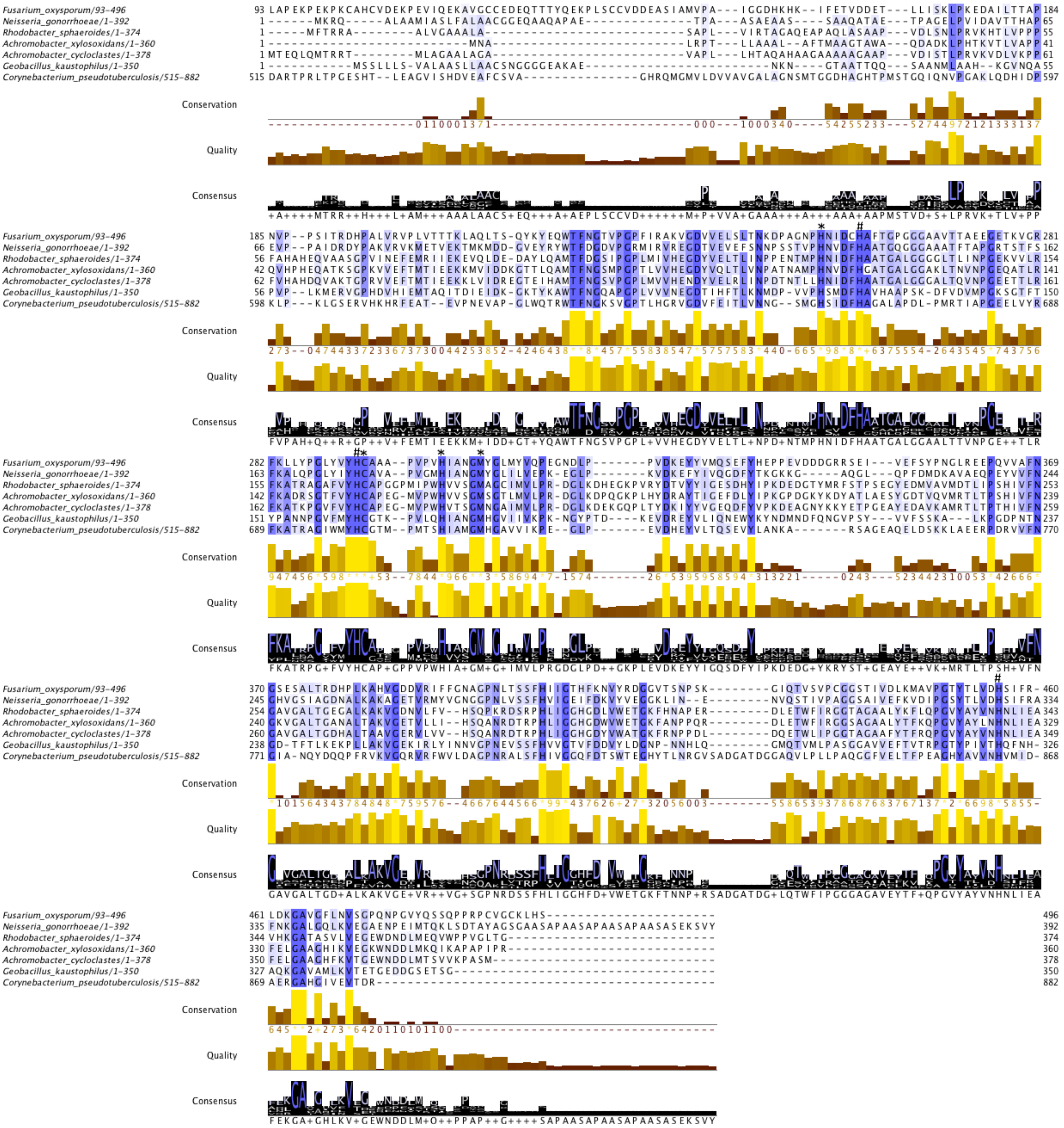

S6 Figure. Amino acid sequence alignment of bacterial NirKs.

Multiple alignment, the program used is ClustalO. Shading highlights amino acid residues of bacterial CuNiRs colored by percentage identity. Putative Cu-binding residues for type 1 and type 2 Cu are indicated by * and #, respectively. Accession numbers of the sequences shown here are as follows: *F. oxysporum* (EF600898); *N. gonorrhoeae* (GenBank M97926),*R. sphaeroides* (Rs) (GenBank U62291), *A. xylosoxidans* (EMBL AF051831), and *A. cycloclastes* (GenBank Z48635), *G. kaustophilus* (GenBank GAD15255.1). In B) Similarity sequence analysis between *nirk* from *C. pseudotuberculosis* and bacterial CuNiRs.

Table S1 – Similarity sequence analysis of *nirK* gene of *C. pseudotuberculosis* and bacterial CuNiRs

C. pseudotuberculosis **Protein**	**Species**	**Lenght (aa)**	**ID (%)**	**Similarity (%)**
Nitrite reductase enzyme likely dependent on copper (CuNIR) (*nirK*) - 882aa	*Fusarium oxysporum*	375	32	48
	Neisseria gonorrhoeae	392	33	50
	Rhodobacter sphaeroides	355	32	44
	Achromobacter xylosoxidans	360	30	44
	Achromobacter Cycloclastes	340	32	46
	Geobacillus kaustophilus	351	35	51
	Corynebacterium durum	867	40	54
	Corynebacterium vitaeruminis	845	40	54

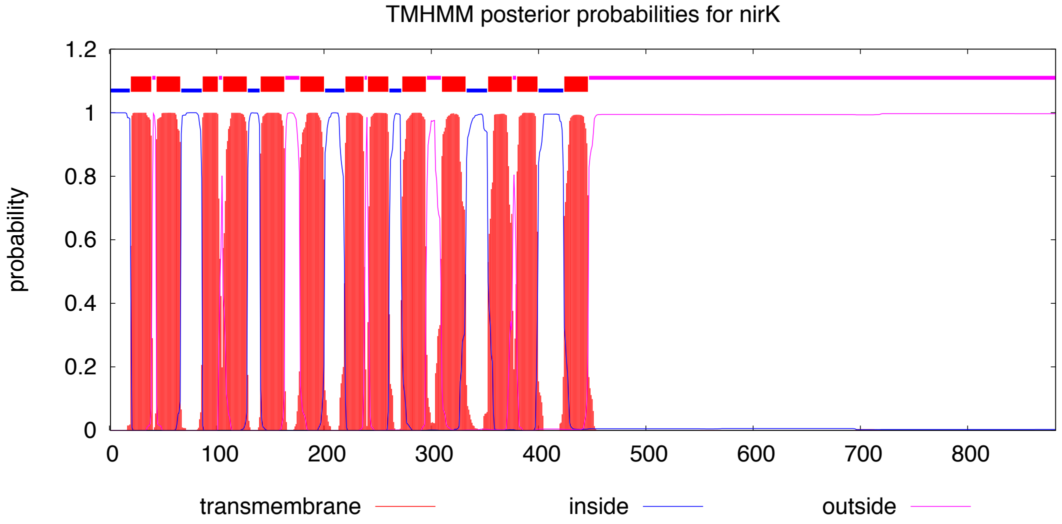

S7 Figure. Transmembrane helices in nirK protein from *C. pseudotuberculosis* biovar Equi.
NirK presented 13 transmembrane helices. Prediction was performed with TMHMM 2.0 (http://www.cbs.dtu.dk/services/TMHMM/).

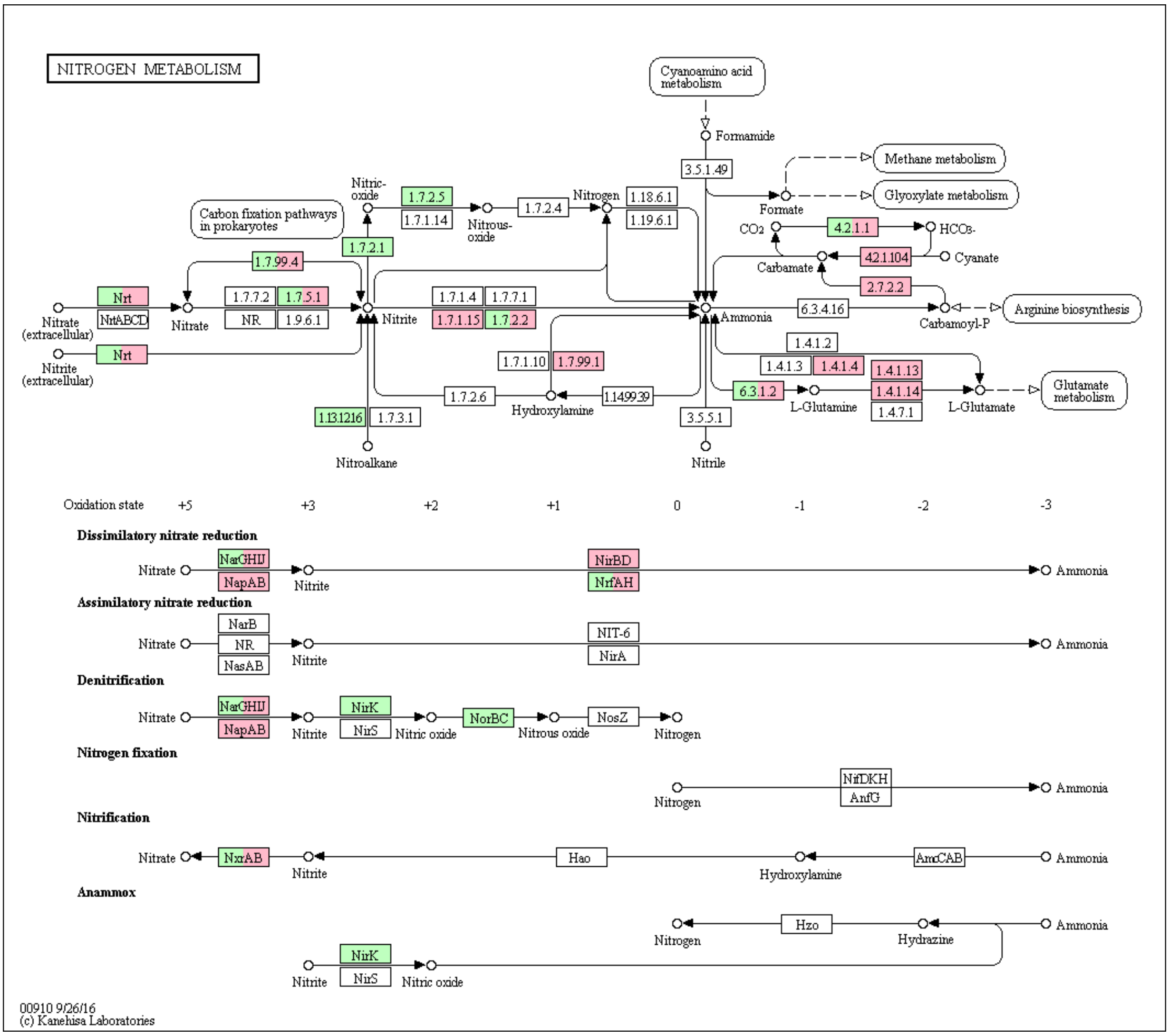

S8 Figure. Comparative nitrogen metabolism between *E.coli* and *C. pseudotuberculosis* biovar Equi. In red pathway to *E. coli*, in green to *C. pseudotuberculosis*. Prediction was performed with KEGG (http://www.genome.jp/kegg/).
